# The Week's Work

**Published:** 1910-07-30

**Authors:** 


### The Week's Work.

### DREADNOUGHT SEAMEN'S HOSPITAL.

We congratulate the committee of the Dreadnought Seamen's Hospital upon receiving the patronage of King George V., accompanied as it is by an intimation that his Majesty has decided to double his annual subscription. This action of his Majesty, coming as it does in the week during which he has devoted himself to a personal inspection of the practical work of many departments of the Navy, proves once more the abiding interest, based upon personal experience and full knowledge, which King George V. takes in the welfare of sailors, whether they be connected with the Royal Navy or with the mercantile fleet. We hope that the example of his Majesty may be followed by every Governor of the Dreadnought Hospital who has the means to enable him to double his subscription during the current year. In this way everyone connected with sailors, and many landsmen who take an intelligent interest in their welfare, may by combination place the Dreadnought Hospital, Greenwich, in a position to do the work which is required to meet fully the need of our seamen in the day of sickness and accident.

### VOLUNTARY HOSPITALS AND MUNICIPAL AUTHORITIES.

An interesting table has been prepared by the authorities of the Bolton Infirmary showing the contributions given to various voluntary hospitals by the municipal authorities. At the Manchester Royal Infirmary the Corporation contributes in subscriptions and police fees £368 13s. per annum; the Salford Royal Hospital received in 1909 £50 with a grant of £200 to the building fund; the Manchester Children's Hospital receives £150; the Western Infirmary, Glasgow, receives £227 3s., including £166 13s. from the trams; the Royal Infirmary, Glasgow, receives £411, including £300 from trams and £70 from gas; the Oldham, Preston, Rochdale, Bury, Blackburn, and Ashton-under-Lyne Voluntary Hospitals all receive subscriptions from the Corporations varying from £131 per annum at Ashton-under-Lyne to £42 at Rochdale. In addition to these subscriptions and grants the Corporations grant the voluntary hospitals a rebate on the water and gas in some cases. Thus at Oldham, Preston, and Blackburn the Corporations supply the water to the hospitals free. At the Western and Royal Infirmaries, Glasgow, the Corporation supplies water and gas at half-price; at the Manchester Royal Infirmary the Corporation makes a reduction in its charge for water, gas, and electricity; the same appears to be done at Salford; whilst at the Manchester Children's Hospital the water is supplied at 3d. per thousand gallons. These facts cannot fail to be of interest to the managers of voluntary hospitals all over the country. They show that in the North of England, at any rate, the municipal authorities do recognise the value of the work done by voluntary hospitals to the whole community, and as we pointed out on a recent occasion, some of the greater Corporations in the North contribute liberally to the building funds of voluntary hospitals.

### THE SUFFRAGIST MEETING IN HYDE PARK.

Bearing insignia the design of which showed, it must be confessed, that lack of breadth and scope characteristic of much feminine work, some thousands of suffragists marched to Hyde Park last Saturday. At the "University" platform were several medical speakers, all of them very well received by the audience. They were Dr. Flora Murray (chairman), Mr. Mansell Moullin (who stated that ninety-eight per cent. of all women graduates in medicine signed a petition in favour of the recent Woman Suffrage Bill), Dr. Helen Fraser, and Sir Victor Horsley. The last named, in a characteristic speech in the course of which he described compromise as "unfortunately a national habit," claimed the policy of the University of London, in admitting women to its degrees for the last thirty years, as a forerunner of the present demonstration. A further step was taken when in 1892 the British Medical Association partially rescinded its old resolution excluding women practitioners from some of the usual privileges of the profession. The community at large, he continued, was only too glad to employ women doctors, but on sweating terms. As an instance, there was a certain Poor Law appointment, recently boycotted by the medical men of the neighbourhood. The authorities appointed a woman, getting her to sign an agreement to retain office in face of any eventuality. When it came to her knowledge, however, that the British Medical Association disapproved of the rate of remuneration, she showed her sense of solidarity by resigning and risking a legal action being taken against her. Prison reform, he added, had been largely a medical question. Little else of professional interest was said, and Sir Victor Horsley concluded by submitting a suitable resolution to the meeting.

### SUFFRAGISM AND HOSPITALS.

Anti-suffragists have busied themselves lately in describing many possible bad effects of the extension of female political power. One small detail seems, however, to have escaped them for the time being, and that is the management of charitable institutions for the sick. Perhaps it is not such a small matter after all. Two things that have been going on side by side for some time are a rapid improvement in surgery and a tendency on the part of the working classes to combine for more and more objects. As a result, in some industrial districts the work of general hospitals has increased by as much as 40 per cent. in the last three or four years; and correspondingly the direction of these institutions has passed to a considerable extent into the control of the classes named. From the strongly democratic note sounded by most pro-suffragist advocates, and from the widespread and, to a considerable extent, justifiable opinion among the laity that the care of the sick is a matter in which women should have a large say, one cannot doubt that female enfranchisement would be likely before long to increase the number of women governors of hospitals. Would this affect adversely the present salutary management of these institutions; would it, for instance, disturb the present admirable loyalty of the nursing to the medical staff? Judging by the recent meeting it could not: matters would go on much the same. At this "University" platform the female speakers certainly charmed their—principally male—audience, acting on the sex-feeling in its subconsciousness. But the real weight of advocacy lay with the men who addressed the meeting. A matter we should have been glad to have heard Sir Victor Horsley's views on is the increased power that would probably accrue to clericalism were women given the vote. Already it is fairly great. It is rather hard, for instance, to see why for every alienist mentioned in connection with care of the feeble-minded there should be ten bishops.

### WORKERS' HOSPITAL FUNDS.

We have to thank Mr. Richard Cole, the Secretary of Addenbrooke's Hospital, Cambridge, for sending us a copy of the rules of the Workers' Hospital Fund, which has been just started in the town and district. Its object is one which is well understood everywhere, but its methods are more popular in the hospitals of the northern than in those of the southern counties, and it is for the benefit of the latter that we discuss them here. The scheme is one for securing regular collective subscriptions from every worker in the neighbourhood, and does not confine itself to any particular class. The northern hospitals have gained regular small sums from each member of the industrial classes which throng their cities for some years past, but it is a new idea to apply the same system to the retail businesses of the south. Here, the populations of the towns being smaller, the fund is made to embrace the district as well. The way in which this is done can be illustrated from the rules drawn up at Cambridge. First, however, it must be realised that the effort is quite voluntary, and that the committee and officers of the fund are controlled and appointed by the workers themselves. One of the rules provides that part of the money subscribed shall be used to send subscribers to convalescent homes, for this has been found valuable at Leeds and Leicester, on the experience of which the Cambridge scheme is modelled. The bulk of the money will be collected by the weekly contributions of the workers, one penny a week from each earner of ten shillings or more, and one halfpenny a week from each earner of a smaller sum. Of the total raised seven-tenths is to be given to Addenbrooke's, and the remaining three-tenths to convalescent homes or other hospitals. Each centre of collection, for every five pounds subscribed annually, will be able to send one delegate to the general meetings, but no centre is to be allowed to have more than two representatives on the board of management. In return for these subscriptions, each centre is to receive recommendations for Addenbrooke's to the amount of seven-tenths of its total contributions, and a further share, equal to the amount of three-tenths of its total contributions, for a convalescent home. The novelty resides not in the above details but in applying the principle which underlies them to other than what is called loosely a factory population. It is a plan worth considering for every southern hospital, and we look forward to seeing the hope of its authors at Cambridge realised, that £1,000 will be raised at the end of the first year, and in process of time three or four times that amount. It is fitting that a University town should be the pioneer of this movement in the south.

### THE DRUG MARKET.

Several further changes in the value of drugs have taken place this week, and there has been a slight improvement in business. Makers of codeine have again reduced their prices by 5d. per oz., and morphine is also tending lower as a result of the lower prices now ruling for opium. Quicksilver has been reduced 2s. 6d. per bottle, but at the time of writing no reduction had been made in the price of calomel and other salts of mercury. Chloral hydrate is again slightly lower in price. Senega is advancing. Menthol is again dearer. Ergot is still tending higher in price.

